# Crafting work with AI: human–AI collaborative job crafting, human–AI fit, and employee job performance

**DOI:** 10.3389/fpsyg.2026.1903517

**Published:** 2026-08-14

**Authors:** Jun Bao, Qiutong Wang, Yiting Pan, Xiaoqian Wang, Yuying Zheng

**Affiliations:** 1 School of Tourism, Huangshan University, Huangshan, China; 2 School of Business Administration, Zhongnan University of Economics and Law, Wuhan, China

**Keywords:** employee job performance, human–AI collaborative job crafting, human–AI fit, inclusive human resource practices, socio-technical systems theory

## Abstract

**Introduction:**

Drawing on sociotechnical systems theory, this study examines how and when human–AI collaborative job crafting is associated with employee job performance.

**Methods:**

Using three-wave data from 485 employees and their direct supervisors, we tested a moderated mediation model involving human–AI fit and inclusive human resource practices.

**Results:**

Human–AI collaborative job crafting was positively associated with human–AI fit, which in turn was associated with higher job performance. Inclusive human resource practices strengthened the positive relationship between human–AI collaborative job crafting and human–AI fit, thereby amplifying its indirect effect on job performance.

**Discussion:**

These findings extend research on job crafting, human–AI fit, and inclusive HR practices, and offer practical implications for organizations seeking to improve employee performance through effective human–AI collaboration.

## 1 Introduction

Artificial intelligence (AI), algorithmic systems, and intelligent devices are becoming increasingly embedded in organizational work. As a result, employees are no longer performing their jobs solely through interpersonal coordination; rather, they are increasingly required to collaborate with AI-enabled systems in task execution, information processing, decision support, and performance evaluation. These developments are reshaping employee behavior and organizational processes across multiple levels (Bankins et al., 2024), while also making work design a central issue in contemporary management research (Parker and Grote, 2022). Human–AI collaboration offers employees opportunities to improve work efficiency, enhance decision quality, and extend the scope of their capabilities. At the same time, it creates new challenges related to technology adaptation, role reconstruction, and work redesign. Employees, however, are not merely passive recipients of technological change. Prior research suggests that employees actively modify their tasks, relationships, cognitions, and resource use to adapt to changing work environments (Parker and Grote, 2022; Tims et al., 2012; Wrzesniewski and Dutton, 2001). In human–AI collaborative work settings, therefore, understanding how employees proactively craft their work around AI-enabled technologies and how such efforts translate into improved job performance has become an important issue in organizational behavior and human resource management.

Existing research has provided valuable insights into the effects of intelligent technologies on employee behavior and performance. For example, task–technology fit theory suggests that technology enhances individual performance when its functions are well aligned with task requirements (Goodhue and Thompson, 1995). The person–task–technology fit framework further indicates that the alignment among individuals, tasks, and technologies shapes technology adoption, technology use, and performance outcomes (Ammenwerth et al., 2006). More recent studies have examined how organizational AI adoption influences employee job crafting (Cheng et al., 2023), how AI crafting affects employees’ AI engagement and helping behavior (Li et al., 2024), and how digital job crafting enables employees to improve person–task–technology fit and job performance by reshaping digital tasks, relationships, cognitions, and technology use (Shi et al., 2023). Despite these advances, three important gaps remain. First, research on AI and human–AI collaboration has focused primarily on technology adoption, algorithmic management, and organizational-level digital transformation, while paying relatively limited attention to employees’ proactive behavioral responses as a source of performance improvement. Second, although job crafting research has been widely developed in traditional work contexts, it has not yet fully explained how employees redesign their work in collaboration with intelligent technologies. Third, the mechanism through which employees’ proactive adjustment to AI-enabled work leads to job performance remains insufficiently understood, particularly from the perspective of human-AI fit.

To address these gaps, this study draws on sociotechnical systems theory to examine how human–AI collaborative job crafting influences employee job performance. Sociotechnical systems theory argues that organizational effectiveness depends not only on the sophistication of technical systems or the capabilities of individual employees, but also on the joint optimization of social and technical systems (Clegg, 2000; Emery and Trist, 1960). Building on this perspective, this study conceptualizes human–AI collaborative job crafting as employees’ overall self-initiated efforts to adjust their work methods, AI-use strategies, and collaboration patterns in order to improve the effectiveness of human–AI collaboration. In human–AI collaborative settings, employees may, for example, adjust how they allocate work between themselves and AI, refine how they use AI tools, seek support for AI-related work, or reinterpret AI as a work resource. Through such overall proactive adjustment, employees are more likely to achieve a higher level of human–AI fit. When employees perceive that AI-enabled technologies fit their abilities, work needs, and task requirements, such technologies are more likely to function as valuable work resources that improve efficiency, work quality, and goal attainment. Accordingly, this study proposes human-AI fit as a key mediating mechanism linking human–AI collaborative job crafting to employee job performance.

This study further examines the boundary role of inclusive human resource practices. The extent to which human–AI collaborative job crafting can be transformed into human-AI fit depends not only on employees’ proactive efforts, but also on the organizational context in which such efforts occur. Inclusive HR practices provide employees with fair opportunities, diverse training, empowerment, psychological safety, and differentiated support. These practices may help employees learn new technologies, express technology-related needs, participate in work redesign, and experiment with new forms of human–AI collaboration. Prior research suggests that inclusive HRM can mitigate the potential negative effects of workforce diversity and foster learning-oriented behavior (Liu et al., 2023), that age-inclusive HR practices can enhance work engagement by creating a supportive organizational climate (Fan et al., 2023), and that inclusive HRM can strengthen employees’ sense of inclusion by addressing both organizational and individual needs in nonstandard employment relationships (van den Groenendaal et al., 2023). Extending this line of research to AI-enabled work, this study argues that inclusive HR practices strengthen the positive relationship between human–AI collaborative job crafting and human-AI fit. Under a more inclusive HR practices, employees are more likely to receive the resources, support, and psychological safety needed to align their capabilities and work requirements with AI-enabled technologies.

**Figure 1:**
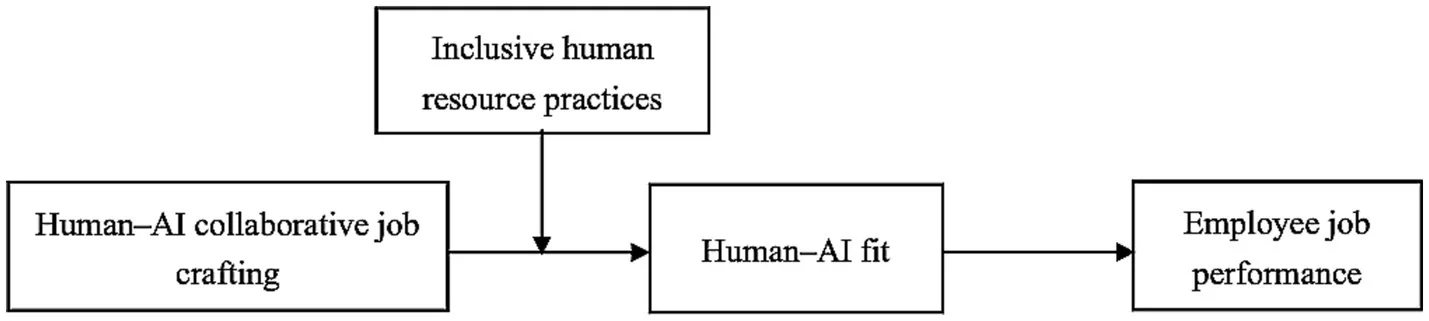
Theoretical model.

This study makes three main theoretical contributions. First, this study extends prior research on digital job crafting to the more specific context of human–AI collaboration. Building on Shi et al. (2023), we focus on AI-enabled work settings in which employees interact with intelligent technologies during task execution, decision support, and work coordination, thereby further revealing how employees’ overall proactive adjustment to AI-enabled work is associated with human–AI fit and job performance. Second, by integrating sociotechnical systems theory with the human-AI fit perspective, this study identifies an indirect association between human–AI collaborative job crafting and employee job performance through human–AI fit. In doing so, it highlights human-AI fit as a key bridge between employee proactivity and performance outcomes in intelligent work environments. Third, this study identifies inclusive HR practices as an important organizational boundary condition, showing how supportive and inclusive management practices enable employees to translate human–AI collaborative job crafting into better fit with intelligent technologies. Practically, this study suggests that organizations should not focus solely on technological investment and process redesign when implementing AI. They should also cultivate employees’ proactive crafting behaviors, strengthen human-AI fit, and develop inclusive HR practices that support effective human–AI collaboration and improved job performance (see Figure 1).

## 2 Literature review and hypotheses

### 2.1 Sociotechnical systems theory

Sociotechnical systems theory originated from the Tavistock research tradition. Its central premise is that organizations are open systems composed of interdependent social and technical elements, including technologies, human actors, task structures, organizational arrangements, and external environments. In their classic study of the longwall coal-mining method, Trist and Bamforth (1951) showed that technological change does not merely alter production processes; it also reshapes collaboration patterns, role structures, and employees’ psychological experiences. When changes in the technical system disrupt existing social relationships and work group structures, both organizational performance and employee adaptation may be impaired. Building on this insight, Emery and Trist (1960) formally advanced the concept of sociotechnical systems and emphasized the interdependence between social and technical systems in organizational design.

From a sociotechnical perspective, technology does not automatically generate performance improvement. Superior organizational outcomes emerge only when technological arrangements, work tasks, employee capabilities, and organizational support are jointly optimized. Subsequent research has further developed sociotechnical systems theory as an important foundation for organization design, information systems implementation, and the management of emerging forms of work. For example, Clegg (2000) argued that sociotechnical principles are applicable to the design of systems involving new information technologies, modern management practices, and changing work arrangements. Similarly, Mumford (2006) and Baxter and Sommerville (2011) highlighted the continuing relevance of sociotechnical thinking for understanding how social and technical components should be integrated in organizational systems.

In the context of human–AI collaboration, sociotechnical systems theory provides a useful lens for explaining how employees and intelligent technologies jointly shape work outcomes. AI-enabled systems are not isolated technical tools; rather, they are embedded in employees’ task execution, decision-making, communication, and performance evaluation. Employees’ overall proactive adjustment to AI-enabled work reflects their efforts to improve alignment between human actors and AI-enabled technical systems. Human-AI fit reflects the extent to which such alignment is achieved, whereas employee job performance represents an important outcome of this alignment. In addition, inclusive human resource practices constitute a key organizational condition that supports employees’ adaptation to AI-enabled work by providing training, participation opportunities, fair treatment, empowerment, and psychological safety. Accordingly, sociotechnical systems theory offers the theoretical foundation for examining how human–AI collaborative job crafting affects employee job performance through human-AI fit, and when this process is strengthened by inclusive HR practices.

### 2.2 Human–AI collaborative job crafting and employee job performance

Human–AI collaborative job crafting refers to employees’ overall proactive adjustment of their work in contexts where they interact and collaborate with AI-enabled systems, algorithmic technologies, or intelligent machines. This construct captures a general tendency to modify work methods, AI-use strategies, and collaboration patterns so as to make human–AI collaboration more effective. Job crafting research suggests that employees are not merely passive recipients of predefined job designs. Instead, they actively reshape their work tasks, relationships, and perceptions of work to better align their work with their motives, abilities, and values (Berg et al., 2013; Wrzesniewski and Dutton, 2001). From the job demands–resources perspective, Tims et al. (2012) further argued that job crafting reflects employees’ proactive modification of job resources and job demands to achieve personal work goals and improve work experiences. Consistent with this perspective, Bakker et al. (2012) showed that employees who proactively craft their jobs are more likely to remain engaged and achieve higher job performance, suggesting that proactive work adjustment can generate important motivational and performance-related outcomes.

In digital and intelligent work settings, technology is no longer a peripheral tool but is deeply embedded in task processes, communication, collaboration, and decision-making. As a result, employees need to proactively adjust not only what they do, but also how they use technologies, how they collaborate with intelligent systems, and how they define the boundaries between human and AI contributions (Parker and Grote, 2022). Building on this view, this study conceptualizes human–AI collaborative job crafting as a behavioral process through which employees proactively optimize human–AI collaboration by crafting tasks, relationships, cognition, and technology use in AI-enabled work settings.

Human-AI fit refers to the degree of compatibility between employees’ individual characteristics and the characteristics of the technologies they use. It reflects whether employees’ knowledge, skills, experience, needs, and work preferences are aligned with the functionality, usability, complexity, and application requirements of technology. Task–technology fit theory argues that information technology improves individual performance when its functions effectively support task completion (Goodhue and Thompson, 1995). Extending this logic, the person–task–technology fit framework suggests that technology adoption and use depend on the fit among individual characteristics, task characteristics, and technology characteristics (Ammenwerth et al., 2006). Technostress research also indicates that misfit between technology characteristics and individual abilities or needs may trigger stress responses and negative behavioral outcomes, whereas higher human-AI fit enables employees to understand, accept, and use technologies more smoothly (Ayyagari et al., 2011). Accordingly, this study defines human-AI fit as the extent to which employees’ technological capabilities, usage needs, and work preferences fit the functions, operating modes, and application requirements of intelligent technologies in human–AI collaborative work.

Human–AI collaborative job crafting may enhance human–AI fit because employees who actively adjust their work around AI-enabled systems are more likely to reduce misalignment between their capabilities, work needs, and technological functions (Parker and Grote, 2022). For example, employees may refine how they divide work between themselves and AI, experiment with more effective AI-use strategies, seek advice or feedback when encountering AI-related difficulties, and reinterpret AI as a complementary work resource rather than merely as a source of uncertainty (Raisch and Krakowski, 2021). These behaviors reflect an overall proactive effort to improve the alignment between employees and intelligent technologies. Therefore, human–AI collaborative job crafting is expected to improve employees’ fit with AI-enabled systems (Goodhue and Thompson, 1995; Kristof-Brown et al., 2005).

According to the person–task–technology fit framework, proactive adjustments in individuals, tasks, and technologies can improve the fit among these elements (Ammenwerth et al., 2006). Therefore, human–AI collaborative job crafting is expected to improve employees’ fit with intelligent technologies by fostering overall proactive adjustment to AI-enabled work. Accordingly, this study proposes:

*Hypothesis 1*: Human–AI collaborative job crafting is positively related to human-AI fit.

### 2.3 Human-AI fit and employee job performance

Employee job performance is a core outcome variable in organizational behavior and human resource management. It generally refers to employees’ work-related behaviors and outcomes that contribute to organizational functioning and goal attainment over a given period. Classic performance research emphasizes that job performance is not limited to final outputs; rather, it is behavioral, evaluative, and multidimensional, reflecting the extent to which employees’ role-related behaviors contribute to organizational objectives (Motowidlo et al., 1997). More specifically, employee job performance may include task performance, contextual performance, and counterproductive work behavior. Task performance reflects the efficiency and quality with which employees fulfill core job responsibilities; contextual performance captures behaviors that support the social and psychological environment of the organization; and counterproductive work behavior undermines organizational goals (Rotundo and Sackett, 2002). Campbell and Wiernik (2015) also noted that individual job performance is one of the most important dependent variables in organizational psychology and organizational behavior. In this study, employee job performance in human–AI collaborative work refers to employees’ behaviors and outcomes that contribute to task accomplishment, work quality, organizational functioning, and goal attainment.

This study argues that higher human-AI fit leads to better employee job performance. Prior person–environment fit research has shown that fit is consistently associated with important individual-level outcomes, including job performance (Kristof-Brown et al., 2005). Task–technology fit theory suggests that the performance value of information technology depends on the extent to which its functions fit task requirements. When technology effectively supports task completion, technology use is more likely to translate into performance gains (Goodhue and Thompson, 1995). The person–task–technology fit framework further emphasizes that technology adoption and use depend not only on the technology itself, but also on the alignment among individual characteristics, task characteristics, and technology characteristics (Ammenwerth et al., 2006).

In human–AI collaborative work, high human-AI fit means that employees’ digital skills, technological experience, usage needs, and work preferences are well aligned with the functions, complexity, and usage requirements of intelligent technologies. Under such conditions, employees can more effectively understand, access, and integrate AI-enabled technologies into work processes. This reduces operational friction and cognitive burden while improving task efficiency, decision quality, and work accuracy. By contrast, when employees’ abilities, needs, or preferences are misaligned with technology characteristics, intelligent technologies may create work overload, role ambiguity, and technostress, ultimately undermining job performance (Ayyagari et al., 2011). Accordingly, this study proposes:

*Hypothesis 2*: Human-AI fit is positively related to employee job performance.

### 2.4 The mediating role of human-AI fit

Sociotechnical systems theory emphasizes that organizational performance depends on the joint optimization of social and technical systems, rather than on technological sophistication or employee capabilities alone (Clegg, 2000; Emery and Trist, 1960). In human–AI collaborative work, employees’ proactive adjustment represents an important behavioral process through which they align themselves with intelligent technologies.

Human-AI fit explains how human–AI collaborative job crafting is translated into employee job performance. By crafting their work around AI-enabled systems, employees can better match their capabilities, work needs, and usage preferences with the functions and requirements of intelligent technologies. Such fit reduces technology-related friction and cognitive burden, while enabling employees to use AI more effectively to improve efficiency, work quality, and decision accuracy. Accordingly, human–AI collaborative job crafting is expected to enhance job performance by improving human-AI fit. Thus, this study proposes:

*Hypothesis 3*: Human-AI fit mediates the positive relationship between human–AI collaborative job crafting and employee job performance.

### 2.5 The moderating effect of inclusive human resource practices

Inclusive human resource practices refer to HR practices that embed the principle of inclusion into recruitment and selection, training and development, job assignment, performance appraisal, compensation, and employee participation. Such practices emphasize respect for employee differences, equal opportunities, diverse development, employee voice, and involvement in organizational decision-making. Prior research suggests that inclusive HR practices help organizations manage workforce diversity, reduce the potential risks of conflict arising from differences, and foster a fair, supportive, and psychologically safe work environment that enables employees to learn, participate, and develop their potential (Ayoko and Fujimoto, 2023; Liu et al., 2023). Recent studies further show that inclusive HRM is not limited to traditional employment relationships but can also be applied to flexible and nonstandard employment arrangements, where clear communication, expectation negotiation, and organizational support facilitate individuals’ inclusion in the organizational system (van den Groenendaal et al., 2023).

Drawing on sociotechnical systems theory, employees’ adaptation in human–AI collaborative contexts depends not only on their proactive adjustment, but also on whether the organizational social system provides supportive conditions for the integration of technical systems. Human–AI collaborative job crafting reflects employees’ overall proactive adjustment to AI-enabled work. However, whether such proactive crafting can be translated into human-AI fit is shaped by organizational HR practices.

When inclusive HR practices are strong, organizations are more likely to provide technology-related training, opportunities for participation, room for trial and error, fair evaluation, and cross-functional support. These practices can reduce employees’ anxiety, defensiveness, and uncertainty toward AI technologies while enhancing their willingness to learn new technologies, express technology-related needs, and experiment with new forms of human–AI collaboration. In such an inclusive environment, employees’ proactive crafting efforts are more likely to result in a better fit between their capabilities, work needs, and intelligent technologies.

By contrast, when inclusive HR practices are weak, employees may lack sufficient resources, psychological safety, and developmental opportunities to adapt to AI-enabled work. Even if employees attempt to craft their tasks, technology use, or collaboration patterns, these efforts may not effectively improve human-AI fit because employees may have limited access to training, insufficient voice opportunities, or concerns about making mistakes when experimenting with AI-enabled tools. Recent research on AI and work design also indicates that the impact of AI on employees is not determined by technology alone, but depends on work design, organizational support, employee attitudes, and human–AI interaction patterns (Bankins et al., 2024; Parker and Grote, 2022). Therefore, inclusive HR practices are expected to strengthen the relationship between human–AI collaborative job crafting and human-AI fit. Accordingly, this study proposes:

*Hypothesis 4*: Inclusive human resource practices moderate the relationship between human–AI collaborative job crafting and human-AI fit, such that the positive relationship is stronger when inclusive human resource practices are high rather than low.

### 2.6 The moderated mediation effect

Integrating the mediating role of human-AI fit and the moderating role of inclusive human resource practices, this study further proposes a moderated mediation effect. Human–AI collaborative job crafting enhances employee job performance by improving human-AI fit. However, this indirect effect depends on the extent to which the organization provides inclusive HR practices. When inclusive HR practices are strong, employees are more likely to receive training, support, psychological safety, and participation opportunities, which helps transform their proactive crafting efforts into better human-AI fit and, subsequently, higher job performance. By contrast, when inclusive HR practices are weak, the indirect effect of human–AI collaborative job crafting on job performance through human-AI fit is likely to be weaker. Accordingly, this study proposes:

*Hypothesis 5*: Inclusive human resource practices moderate the indirect relationship between human–AI collaborative job crafting and employee job performance through human-AI fit, such that this indirect relationship is stronger when inclusive human resource practices are high rather than low.

## 3 Materials and methods

### 3.1 Sample selection and data sources

This study adopted a three-wave time-lagged research design to reduce potential common method bias. Data were collected from employees and their direct supervisors in six AI service companies in central China. These companies were operated in AI-enabled service domains such as intelligent customer service, AI-assisted data analytics, enterprise AI solutions, and algorithm-based service support. The participating companies varied in size, ranging from approximately 800 to 1,500 employees. Employees in these firms regularly interacted with AI-enabled systems in their daily work, including AI-assisted customer response systems, algorithmic decision-support tools, and intelligent data-processing platforms. At Time 1, we distributed 521 questionnaires to employees to collect self-reported data on inclusive human resource practices and human–AI collaborative job crafting, as well as control variables and demographic characteristics. A total of 505 questionnaires were returned. At Time 2, 2 weeks after Time 1, we distributed questionnaires to the same employees to measure human–AI fit, and 492 questionnaires were returned. At Time 3, 2 weeks after Time 2, we invited the direct supervisors of the same employees to evaluate their job performance. Finally, 485 valid employee–supervisor matched responses were obtained, yielding an effective response rate of 93.1%. The two-week interval was chosen to balance substantive and practical considerations, allowing sufficient time for employees to adjust their work methods and form initial perceptions of human–AI fit while minimizing memory decay and participant attrition. Participants received a nominal monetary compensation of RMB 3.00 for completing the survey. Among the 485 valid participants, 46.0% were female, 54.9% were between 26 and 35 years old, 76.9% held a bachelor’s degree or above, and 59.8% had organizational tenure of 5–15 years.

Because employees were recruited from six companies, we calculated ICC (1) values to assess the extent of between-company variance in the focal variables, including human–AI collaborative job crafting, human–AI fit, employee job performance, and inclusive human resource practices. Although there is no fixed cut-off point for ICC(1), prior methodological guidance suggests that values around 0.01 may be interpreted as small effects, values around 0.10 as medium effects, and values above 0.25 as large effects (Murphy and Myors, 1998). The ICC (1) values ranged from 0.02 to 0.06, all below the medium-effect benchmark of 0.10. These results suggest that between-company differences accounted for only a limited proportion of variance in the focal variables. Accordingly, the use of individual-level analyses was considered appropriate.

### 3.2 Measures

All constructs were measured using 5-point Likert scales (1 = disagree, 5 = agree). We followed the standard translation and back-translation procedure to ensure the accuracy of the Chinese versions of the scales (Schaffer and Riordan, 2003). All measurement items are reported in Appendix A. We report Cronbach’s *α* coefficients for each construct to assess reliability.

#### 3.2.1 Human–AI collaborative job crafting

Human–AI collaborative job crafting was measured using a four-item scale adapted from He et al. (2024). Consistent with our narrowed conceptualization, the scale was used to capture employees’ overall proactive adjustment to AI-enabled work. Participants evaluated the extent to which they proactively adjusted their work methods and environment to collaborate effectively with AI systems. The original items were adapted to the AI-enabled work context while retaining the core meaning of proactive work adjustment. To assess content validity, three scholars with expertise in organizational behavior and job crafting reviewed the adapted items for clarity, contextual appropriateness, and consistency with our definition of HACJC. A sample item is: “I introduce new approaches to improve my work when working with AI.” (Cronbach’s *α* = 0.87).

#### 3.2.2 Human–AI fit

Human–AI fit was measured using a five-item scale from Wu (2026). Participants evaluated the compatibility between their work style and the AI functionality and outputs. A sample item is: “Interacting with AI feels natural and intuitive to me.” (Cronbach’s α = 0.82).

#### 3.2.3 Employee job performance

Employee job performance was measured using a 5-item supervisor-rated scale from Janssen and Van Yperen (2004). Supervisors evaluated the extent to which employees fulfilled their core job duties. A sample item is: “This worker always completes the duties specified in his/her job description.” (Cronbach’s α = 0.84).

#### 3.2.4 Inclusive human resource practices

Inclusive human resource practices were measured using a five-item scale from Qu et al. (2024). Participants evaluated the extent to which their organization’s HR practices were inclusive of diverse employee backgrounds. A sample item is: “The company encourages employees with different backgrounds (e.g., gender, ethnicity, religion, native place, dialect, personality, and disability status) to voice their suggestions.” (Cronbach’s α = 0.89).

#### 3.2.5 Control variables

Following previous research, we controlled for several individual-level demographic variables, including gender, age, education level, and organizational tenure, because these variables may influence employees’ work attitudes and performance outcomes (Darvishmotevali and Altinay, 2022). In addition, we controlled for employees’ duration of AI use, as the length of experience with AI may affect their attitudes toward and behavioral responses to AI-enabled work (Mo et al., 2024).

## 4 Results

### 4.1 Common method Bias test

Although the three-wave design and supervisor-rated measure of employee job performance helped reduce potential common method bias, human–AI collaborative job crafting, human–AI fit, and inclusive human resource practices were measured using employee self-report data. Therefore, following Tang and Wen (2020), we conducted Harman’s single-factor test to assess potential common method bias. The results of the exploratory factor analysis showed that the first unrotated factor explained 29.42% of the total variance, which is below the conventional threshold of 40%. However, as Harman’s single-factor test is relatively insensitive (Podsakoff et al., 2003, 2012), we adopted a more stringent approach by introducing an unmeasured latent method factor to further control for potential method effects (Podsakoff et al., 2003). After the inclusion of this factor, the model fit showed minimal improvement (Δχ^2^/df = 0.05, ΔCFI = 0.004, ΔTLI = 0.006, and ΔRMSEA = 0.002). In addition, the common latent factor test showed that changes in the standardized factor loadings attributable to the common latent factor were all below 0.20 (Chin, 1998), suggesting that common method bias was not a serious concern in this study.

### 4.2 Descriptive statistics and confirmatory factor analysis

Table 1 presents the means, standard deviations, and correlations among the study variables and control variables. To examine the distinctiveness of the four focal constructs—human–AI collaborative job crafting, human–AI fit, employee job performance, and inclusive human resource practices—we conducted confirmatory factor analysis using Mplus 8.3.

**Table 1:** Means, standard deviations, correlations, and reliabilities.

Variables	1	2	3	4	5	6	7	8	9
1. Gender	—								
2. Age	−0.09^*^	—							
3. Education	−0.01	−0.02	—						
4. Tenure	−0.12^**^	−0.20^**^	0.03	—					
5. AI time	−0.04	0.03	−0.09^*^	0.02	—				
6. Human–AI collaborative job crafting	−0.03	−0.11^*^	0.10^*^	0.12^**^	0.08	(0.87)			
7. Human–AI Fit	−0.03	−0.15^**^	−0.01	0.05	−0.13^**^	0.36^**^	(0.82)		
8. Employee job performance	−0.05	−0.13^**^	0.02	0.12^*^	−0.04	0.22^**^	0.39^**^	(0.84)	
9. Inclusive human resource practices	−0.02	−0.18^**^	0.09	0.12^**^	0.01	0.25^**^	0.27^**^	0.14^**^	(0.89)
Mean	0.46	1.87	3.57	3.69	2.41	3.66	3.46	3.88	3.33
SD	0.50	0.73	1.46	0.62	1.21	0.60	0.64	0.81	0.71

*N* = 485; *p < 0.05, **p < 0.01; Values in the brackets are Cronbach’s alpha coefficients. Gender was coded female = 1 and male = 0.

As shown in Table 2, the hypothesized four-factor model demonstrated acceptable model fit: χ^2^(146) = 279.76, χ^2^/df = 1.92, CFI = 0.97, TLI = 0.96, RMSEA = 0.04, and SRMR = 0.04. Compared with the alternative competing models, the four-factor model showed the best fit to the data, supporting the discriminant validity of the focal constructs.

**Table 2:** The results of CFA.

Models	*χ* ^2^	df	*χ*^2^/ df	CFI	TLI	RMSEA	SRMR
Four-factor model: HACJC; HAF; EJB; IHRP	279.76	146	1.92	0.97	0.96	0.04	0.04
Three-factor model: HACJC; HAF + EJB; IHRP	832.45	149	5.59	0.84	0.81	0.10	0.08
Two-factor model: HACJC+HAF + EJB; IHRP	1502.39	151	9.95	0.68	0.64	0.14	0.11
Single-factor model: HACJC+HAF + EJB + IHRP	2604.73	152	17.14	0.42	0.34	0.18	0.15

*N* = 485; HACJC, Human–AI collaborative job crafting; HAF, Human–AI fit; EJB, Employee Job performance; IHRP, Inclusive human resource practices.

As reported in Table 3, all standardized factor loadings exceeded 0.60. The composite reliability values for the four constructs were 0.87, 0.83, 0.84, and 0.89, respectively, all exceeding the recommended threshold of 0.70. The average variance extracted values were 0.62, 0.50, 0.52, and 0.63, respectively, meeting or exceeding the recommended criterion of 0.50 and supporting convergent validity. In addition, consistent with the Fornell–Larcker criterion (Fornell and Larcker, 1981), the square root of the AVE for each construct was greater than its correlations with the other constructs. With AVE values ranging from 0.50 to 0.63 and the largest squared inter-construct correlation being 0.15, the results provided further support for discriminant validity. Notably, HAF’s AVE hits the 0.5 cutoff exactly, with low loadings of HAF4 (0.64) and HAF5 (0.56). HAF5’s weak loading suppresses the construct’s convergent validity. Though borderline, the construct meets the Fornell–Larcker criterion (Fornell and Larcker, 1981). Although HAF5 had a relatively low loading, it was retained because it captures the perceived naturalness and intuitiveness of interacting with AI, reflecting the experiential compatibility aspect of human–AI fit. Overall, the HAF construct met the AVE and CR thresholds and satisfied the Fornell–Larcker criterion, supporting acceptable validity.

**Table 3:** First-order confirmatory factor analysis.

Variables	Items	Loadings	AVE	CR
Human–AI collaborative job crafting (HACJC)	HACJC 1	0.78	0.62	0.87
	HACJC 2	0.81		
	HACJC 3	0.79		
	HACJC 4	0.77		
Human–AI Fit (HAF)	HAF 1	0.79	0.50	0.83
	HAF 2	0.78		
	HAF 3	0.71		
	HAF 4	0.64		
	HAF 5	0.56		
Employee job performance (EJP)	EJP 1	0.68	0.52	0.84
	EJP 2	0.76		
	EJP 3	0.77		
	EJP 4	0.73		
	EJP 5	0.66		
Inclusive human resource practices (IHRP)	IHRP 1	0.79	0.63	0.89
	IHRP 2	0.85		
	IHRP 3	0.83		
	IHRP 4	0.82		
	IHRP 5	0.65		

CR, composite reliability; AVE, average variance extracted.

In addition to the Fornell–Larcker criterion, we assessed discriminant validity using the heterotrait–monotrait ratio of correlations (HTMT). As shown in Table 4, the HTMT values ranged from 0.16 to 0.47, all below the conservative threshold of 0.85 (Henseler et al., 2015), providing further evidence of discriminant validity among the focal constructs.

**Table 4:** HTMT ratios.

Variables	HACJC	HAF	IHRP	EJP
HACJC	—			
HAF	0.428	—		
IHRP	0.282	0.312	—	
EJP	0.249	0.465	0.161	—

HACJC, human–AI collaborative job crafting; HAF, human–AI fit; IHRP, inclusive human resource practices; EJP, employee job performance; HTMT, heterotrait–monotrait ratio of correlations; HTMT ratios (good if < 0.90, best if < 0.85).

### 4.3 Hypothesis testing

We used Hayes’ (2013) PROCESS Model 7 to test the mediation and moderated mediation hypotheses. As shown in Table 5, human–AI collaborative job crafting was positively associated with human–AI fit (*b* = 0.37, *p* < 0.001), and human–AI fit was positively associated with employee job performance (*b* = 0.39, *p* < 0.001), supporting Hypotheses 1 and 2. The indirect effect of human–AI collaborative job crafting on employee job performance through human–AI fit was significant, with a bootstrapped indirect effect of 0.15 and a 95% confidence interval of [0.10, 0.20]. The direct effect of human–AI collaborative job crafting on employee job performance was not significant (*b* = 0.08, ns). However, consistent with contemporary mediation guidance (Rucker et al., 2011; Zhao et al., 2010), we did not use the nonsignificance of the direct effect to infer “full mediation.” Rather, these results were consistent with an indirect-only mediation pattern. Accordingly, Hypothesis 3. was supported.

**Table 5:** The results of the moderated mediation model.

Variables	Human–AI fit		Employee job performance	
	b	S.E.	b	S.E.
Control variable				
Gender	−0.03	0.05	−0.05	0.06
Age	−0.07	0.04	−0.06	0.04
Education	−0.01	0.02	0.04	0.02
Tenure	−0.05	0.04	0.01	0.05
AI time	−0.08	0.02	0.01	0.02
Independent variable				
Human–AI collaborative job crafting	0.37^***^	0.04	0.08	0.05
Mediator				
Human–AI fit			0.39^***^	0.05
Moderator				
Inclusive human resource practices	0.15^***^	0.04		
Interaction				
Human–AI collaborative job crafting × Inclusive human resource practices	0.20^***^	0.04		
R^2^	0.23		0.17	
F	18.05^***^		14.13^***^	

*N* = 485; ***p < 0.001.

**Figure 2:**
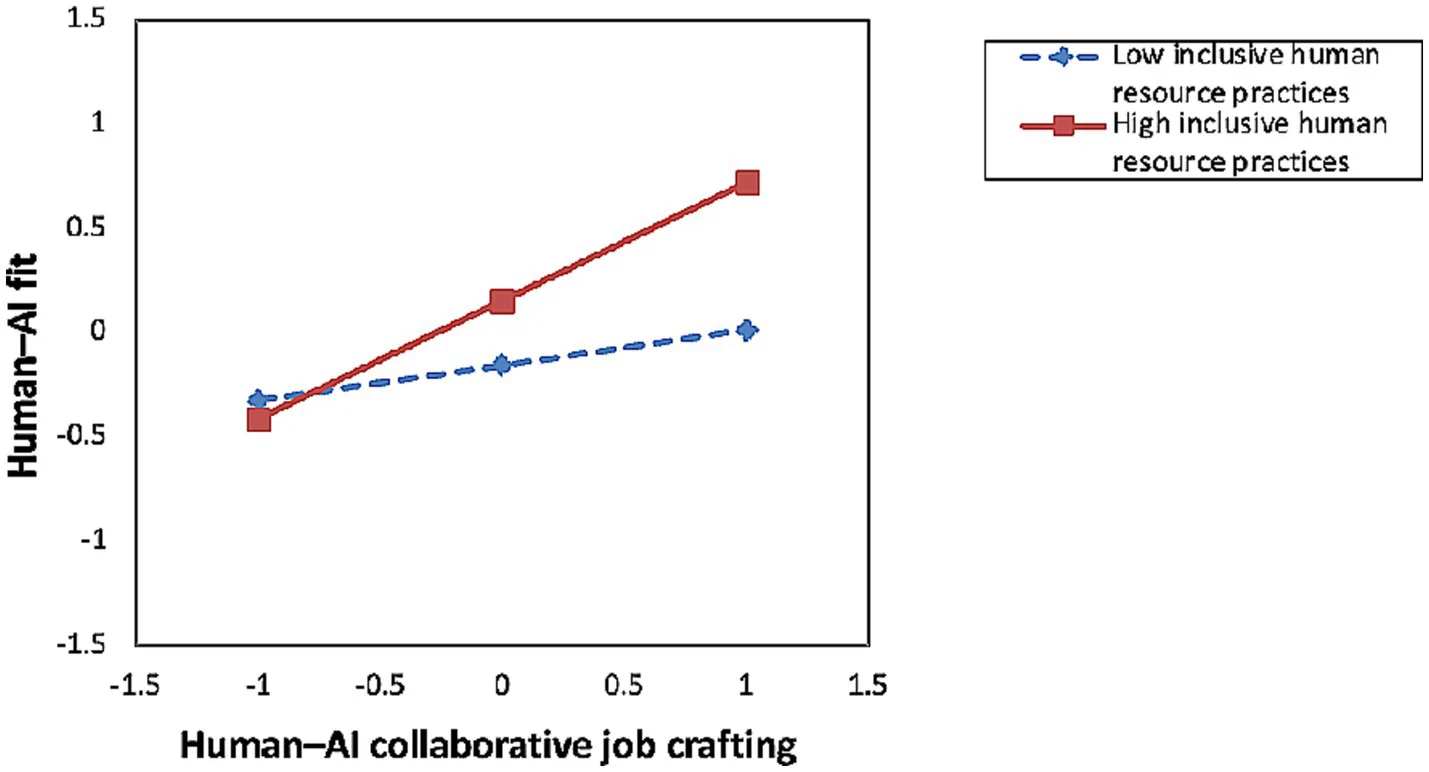
Influences of human–AI collaborative job crafting and inclusive human resource practices on human–AI fit. Both axes represent mean-centered standardized values.

Hypothesis 4 proposed that inclusive human resource practices moderate the relationship between human–AI collaborative job crafting and human–AI fit. The results showed that the interaction between human–AI collaborative job crafting and inclusive human resource practices was significant in predicting human–AI fit (*b* = 0.20, *p* < 0.001). Simple slope analyses further indicated that the relationship between human–AI collaborative job crafting and human–AI fit was stronger when inclusive human resource practices were high (*b* = 0.51, *p* < 0.001) than when they were low (*b* = 0.23, *p* < 0.001). Figure 2 illustrates the interaction effect at one standard deviation above and below the mean of inclusive human resource practices. These results support Hypothesis 4.

Finally, we examined the conditional indirect effect proposed in Hypothesis 5. As reported in Table 6, the index of moderated mediation was significant, with an index value of 0.08 and a 95% confidence interval of [0.04, 0.13]. This result indicates that the indirect effect of human–AI collaborative job crafting on employee job performance through human–AI fit varied as a function of inclusive human resource practices. Further analyses showed that the indirect effect was stronger when inclusive human resource practices were high (indirect effect = 0.20, 95% CI [0.13, 0.28]) than when they were low (indirect effect = 0.09, 95% CI [0.05, 0.15]). Because the focal variables were standardized before estimating PROCESS Model 7, the conditional indirect effects reported in Table 6 were estimated on a standardized scale. Therefore, Hypothesis 5 was supported.

**Table 6:** Results for hypothesized conditional indirect effects.

Condition	Indirect effect	SE	95% confidence interval
Human–AI Collaborative Job Crafting → Human–AI Fit → Employee Job performance			
Inclusive human resource practices (−1SD)	0.09	0.03	[0.05, 0.15]
Inclusive human resource practices (+1SD)	0.20	0.04	[0.13, 0.28]
Index of moderated mediation	0.08	0.02	[0.04, 0.12]

*N* = 485, Bootstrap = 5,000; conditional indirect effects were estimated using standardized focal variables.

## 5 Discussion

Drawing on sociotechnical systems theory and the practical context of AI-enabled organizational work, this study developed and tested a moderated mediation model to explain how human–AI collaborative job crafting influences employee job performance. Based on data from 485 employee–supervisor matched dyads, the findings provide evidence consistent with a mechanism and boundary condition through which employees’ proactive adaptation to intelligent technologies is associated with higher job performance.

First, human–AI collaborative job crafting was positively related to human-AI fit. This finding suggests that employees can better align their capabilities and work demands with the functions of intelligent technologies by proactively adjusting task allocation, optimizing technology use, reconstructing collaborative relationships, and reshaping their cognition of AI. Second, human-AI fit was positively related to employee job performance and mediated the relationship between human–AI collaborative job crafting and employee job performance. This finding indicates that human–AI collaborative job crafting does not necessarily translate directly into improved job performance; rather, it enhances job performance indirectly by increasing employees’ perceived human–AI fit. Third, inclusive human resource practices moderated the relationship between human–AI collaborative job crafting and human-AI fit. Specifically, the positive association between human–AI collaborative job crafting and human–AI fit was stronger when employees reported more inclusive HR practices, thereby strengthening the indirect association between crafting and performance through fit.

### 5.1 Theoretical implications

This study makes several theoretical contributions. First, this study extends prior research on digital job crafting by examining employees’ overall proactive adjustment in the specific context of human–AI collaboration. We acknowledge that Shi et al. (2023) have already proposed digital job crafting and demonstrated its relationship with person–task–technology fit and job performance. Building on this foundation, our study focuses on AI-enabled work, in which employees do not merely use digital tools but also collaborate with intelligent systems in task execution, decision-making, and work coordination. By narrowing the focus to human–AI collaboration, this study clarifies how employees’ proactive adjustment to AI-enabled work is associated with human–AI fit, thereby extending digital job crafting research.

Second, this study advances research on human–AI fit by explaining why and how employees’ proactive adjustment to AI-enabled work is associated with performance-related outcomes. Prior research has established that fit between individuals, tasks, and technologies is important for technology use and job performance (Ammenwerth et al., 2006; Shi et al., 2023). However, less is known about how such fit is actively created by employees in AI-enabled work settings. Our findings show that human–AI collaborative job crafting is positively related to employee job performance through human–AI fit. This suggests that proactive crafting does not necessarily generate performance benefits directly or automatically. Rather, its performance relevance depends on whether employees’ capabilities, work needs, and usage preferences become better aligned with the functions, operating modes, and application requirements of AI systems. Therefore, the key insight is not simply that “fit matters,” but that human–AI fit helps explain the indirect association between employees’ proactive adjustment to AI-enabled work and their job performance. This helps explain why employees working with similar AI technologies may experience different performance outcomes. In addition, the correlation results in Table 1 showed that duration of AI use was negatively related to human–AI fit (*r* = −0.13, *p* < 0.01). Although this finding appears counterintuitive, it suggests that longer exposure to AI-enabled systems does not necessarily lead to better perceived fit. One possible explanation is that extended AI use may make employees more aware of the limitations, complexity, and constraints of AI-enabled systems. This further supports our argument that human–AI fit depends not on exposure alone but on employees’ proactive adjustment and supportive organizational conditions.

Third, this study extends research on inclusive HR practices and sociotechnical systems theory by identifying inclusive human resource practices as a social-system condition that enables employees’ proactive adjustment to translate into human–AI fit. Existing inclusive HR practices research has mainly focused on diversity management, employee inclusion, work engagement, and nonstandard employment relationships (Fan et al., 2023; Liu et al., 2023; van den Groenendaal et al., 2023). Extending this literature, our findings show that inclusive HR practices also play an important role in AI-enabled work by providing employees with support for learning, experimentation, voice, and adaptation. This contribution also refines the application of sociotechnical systems theory at the individual level: joint optimization is not achieved only through organizational design or technology implementation, but also through the interaction between employees’ proactive crafting behavior and an inclusive HR context that supports technology adaptation.

Notably, inclusive HR practices amplify the association between human–AI collaborative job crafting and human–AI fit. This stage-divergent moderating pattern can be explained by the three psychological foundations proposed in hypothesis development: psychological safety, employee voice channels, and organizational tolerance for trial and error. When inclusive HR practices is high, abundant supportive resources make the correlation between crafting behaviors and fit perceptions far stronger (simple slope = 0.51); under weak inclusive HR practices, lack of such resources weakens this linkage substantially (simple slope = 0.23). These findings indicate that an inclusive HR context helps employees translate their proactive crafting efforts into a stronger perceived fit with AI-enabled technologies. In this way, the study shows that effective human–AI collaboration depends on both employee agency and organizational support, thereby offering a more integrated explanation of sociotechnical alignment in AI-enabled workplaces (Bankins et al., 2024; Clegg, 2000; Emery and Trist, 1960; Parker and Grote, 2022).

### 5.2 Practical implications

This study also offers several practical implications. First, organizations should recognize employee proactivity in human–AI collaboration and encourage human–AI collaborative job crafting. AI implementation does not mean that employees should passively adapt to technological arrangements. Rather, organizations should encourage employees to adjust human–AI task allocation, optimize technology use, redesign work processes, and view AI as a valuable resource for improving work efficiency and extending capability boundaries. Managers can use empowerment, feedback, and experience-sharing mechanisms to guide employees in identifying which tasks are more suitable for AI and which tasks require human judgment, creativity, and contextual understanding. Such efforts can improve the effectiveness of human–AI collaboration.

Second, organizations should treat human-AI fit as a key indicator of AI implementation effectiveness. Introducing intelligent technologies alone does not automatically improve employee performance. What matters is whether employees’ capabilities, needs, and work styles fit the functions and application requirements of the technology. Therefore, when promoting AI applications, organizations should strengthen digital skills training, provide clear guidance on technology use, and conduct job–technology fit analysis. These efforts can help employees understand technological functions, master effective usage methods, and match appropriate intelligent tools with different job requirements. For employees with weaker technological capabilities or higher technology anxiety, more targeted support should be provided to prevent AI use from becoming an additional source of burden.

Third, organizations should build a supportive environment for human–AI collaboration through inclusive HR practices. Human–AI collaborative job crafting requires continuous learning, experimentation, and adjustment. Without an inclusive work environment, employees may be reluctant to express difficulties in technology use or to experiment with new forms of human–AI collaboration. Organizations should therefore embed inclusion into recruitment and selection, training and development, performance appraisal, and incentive systems. They should respect differences in employees’ technological capabilities, learning pace, and adaptation styles, while providing fair opportunities, psychological safety, and participation channels. In particular, performance evaluation should not focus solely on technology-use outcomes; it should also recognize employees’ learning effort, adaptation process, and collaborative improvement behaviors, thereby encouraging employees to achieve more effective human-AI fit.

### 5.3 Limitations and future research

This study has several limitations that suggest directions for future research. First, although this study adopted a three-wave, multi-source design to reduce common method bias, the data do not include baseline measures of human–AI fit or employee job performance. Therefore, we were unable to include autoregressive controls for prior human–AI fit or prior performance. In addition, human–AI collaborative job crafting and inclusive human resource practices were both measured at Time 1 using employee self-report data, meaning that the interaction term predicting human–AI fit was constructed from same-source, same-wave measures. These design features limit the extent to which the study can rule out reverse causality, common method concerns, and temporal-ordering concerns. As a result, the findings should be interpreted as lagged associations rather than definitive causal effects. Future research could use longitudinal, experimental, or experience sampling designs and collect inclusive HR practice data from independent sources to examine the dynamic and reciprocal relationships among these variables.

Second, this study examined inclusive human resource practices as a boundary condition. However, other contextual factors, such as digital leadership, organizational learning climate, technology support climate, psychological safety, and AI governance mechanisms, may also influence how employees adapt to intelligent technologies. Future research could investigate these moderators to provide a more comprehensive understanding of the boundary conditions of human–AI collaborative job crafting.

Third, this study mainly focused on the positive effects of human–AI collaborative job crafting. Future research could adopt a double-edged-sword perspective by examining whether excessive work adjustment, frequent technology learning, or overreliance on AI may increase technostress, workload, role ambiguity, or occupational identity threat, thereby undermining employee well-being.

Finally, this study measured human–AI collaborative job crafting as a unidimensional construct reflecting employees’ overall proactive adjustment to AI-enabled work. Although this approach is consistent with our narrowed conceptualization and the adapted four-item scale, it does not allow us to distinguish among potentially different forms of crafting, such as task-related adjustment, technology-use adjustment, relational support seeking, and cognitive reframing. Future research could develop and validate a multidimensional scale of human–AI collaborative job crafting to examine whether these specific forms have distinct antecedents and outcomes.

## 6 Conclusion

This study examined how and when human–AI collaborative job crafting is associated with employee job performance in AI-enabled work settings. Drawing on sociotechnical systems theory, we proposed and tested a moderated mediation model in which human–AI fit serves as the mediating mechanism and inclusive human resource practices serve as the organizational boundary condition. Based on three-wave, multi-source data from 485 employee–supervisor matched dyads, the findings show that employees’ overall proactive adjustment to AI-enabled work is positively related to human–AI fit, which in turn correlates with superior supervisor-rated job performance. The results further demonstrate that inclusive HR practices strengthen the positive indirect effect of human–AI collaborative job crafting on job performance via human–AI fit.

Theoretically, this study expands existing scholarship by extending prior research on digital job crafting to the specific context of human–AI collaboration. Rather than treating employees as passive users of intelligent technologies, this study highlights their proactive role in adjusting work methods, AI-use strategies, and collaboration patterns to achieve better alignment with AI-enabled systems. In addition, this study clarifies the indirect relationship between employees’ proactive adjustment to AI-enabled work and performance-related outcomes through human–AI fit. The study also extends research on inclusive human resource practices by showing that inclusive HR practices can function as an important social-system condition that supports employees’ adaptation to intelligent technologies. From a sociotechnical perspective, these findings suggest that effective human–AI collaboration depends not only on technological capability, but also on the alignment among employees, technologies, work tasks, and organizational support.

Practically, the findings suggest that organizations should not rely solely on technological investment when implementing AI. To improve the effectiveness of human–AI collaboration, managers should encourage employees to actively adjust their work around AI-enabled systems, provide training and feedback that help employees improve human–AI fit, and create inclusive HR practices that support learning, experimentation, voice, and psychological safety. Such practices can help employees better understand AI functions, reduce uncertainty in AI use, and develop more effective ways of integrating intelligent technologies into daily work.

Finally, this study also points to several directions for future research. Although the three-wave, multi-source design strengthens the research design, future studies could use longitudinal, experimental, or experience sampling approaches to further examine the dynamic and reciprocal relationships among human–AI collaborative job crafting, human–AI fit, and employee performance. Future research could also develop multidimensional measures of human–AI collaborative job crafting and explore whether different forms of crafting have distinct antecedents and outcomes. In addition, future studies may examine other contextual factors, such as digital leadership, AI governance, technology support climate, and psychological safety, to provide a more comprehensive understanding of how employees and organizations can jointly optimize human–AI collaboration.

## Data Availability

The raw data supporting the conclusions of this article will be made available by the authors, without undue reservation.

## Appendix A

**Table A:** Measurement items.

Construct	Code	Item
Human–AI Collaborative Job Crafting (HACJC)	HACJC 1	I introduce new approaches to improve my work when working with AI.
	HACJC 2	I change inefficient work procedures when working with AI.
	HACJC 3	I change the way I do my job to make my work easier when working with AI.
	HACJC 4	I rearrange AI-related tools or my working environment to better support my work with AI.
Human–AI Fit (HAF)	HAF 1	The way this AI assistant provides information aligns perfectly with the way I approach my tasks.
	HAF 2	Interacting with this AI assistant feels natural and intuitive to me.
	HAF 3	The functionality of this AI assistant seamlessly integrates into my daily workflow.
	HAF 4	The outputs from this AI assistant match the level of detail and complexity I require for my job.
	HAF 5	Overall, there is an excellent fit between how I work and how this AI assistant supports me.
Employee Job performance (EJP)	EJP 1	This worker always completes the duties specified in his/her job description.
	EJP 2	This worker meets all the formal performance requirements of the job.
	EJP 3	This worker fulfills all responsibilities required by his/her job.
	EJP 4	This worker never neglects aspects of the job that he/she is obligated to perform.
	EJP 5	This worker often fails to perform essential duties.
Inclusive human resource practices (IHRP)	IHRP 1	The company grants employees a certain degree of autonomy in their work.
	IHRP 2	The company’s recruitment and selection decisions are not influenced by candidates’ personality, professional background, or other personal characteristics.
	IHRP 3	The company encourages employees with different backgrounds (e.g., gender, ethnicity, religion, native place, dialect, personality, and disability status) to voice their suggestions.
	IHRP 4	The company provides diversity-related training covering different backgrounds and characteristics (e.g., gender, ethnicity, culture, religion, native place, dialect, personality, and disability status) to promote mutual respect, recognition, and learning among employees.
	IHRP 5	The company widely solicits employees’ opinions when determining performance-based compensation and performance appraisal standards.
